# Silent Type-B Aortic Dissection Accidentally Discovered in a COVID-19-Positive Patient

**DOI:** 10.7759/cureus.41373

**Published:** 2023-07-04

**Authors:** Amr Gebril, Ali Nawaz, Samer Ashour, Mohammed K Nasr, Ossma E Eelbelihy

**Affiliations:** 1 Emergency Medicine, NMC Royal Hospital Khalifa City, Abu Dhabi, ARE; 2 Emergency Medicine, Dr. Sulaiman Al Habib Hospital, Dubai, ARE; 3 Emergency Medicine, Madinat Zayed Hospital, Abu Dhabi, ARE

**Keywords:** conservative management, cardiovascular complications, stanford type b, aortic dissection, covid-19 infection

## Abstract

Aortic dissection is a critical medical condition that poses a significant threat to life, and if left untreated, it can lead to high mortality and morbidity rates. The risk of various cardiovascular complications, including aortic dissection, is increased in individuals with coronavirus disease 2019 (COVID-19). However, the significance of aortic dissection as a complication in COVID-19 patients is often underestimated. Traditionally, aortic dissection without pain was considered uncommon. However, recent information indicates that symptoms in patients with aortic dissection can be more diverse than previously believed. The classic symptoms of tearing chest, back, or abdominal pain may be absent, making diagnosis challenging. We present the incidental detection of an asymptomatic Stanford type-B aortic dissection during a computed tomography (CT) scan conducted to evaluate COVID-19. The patient was managed through conservative treatment.

## Introduction

Aortic dissection is a critical condition that poses a significant risk to life and health if not treated promptly. A total of 27.4% of aortic dissection patients die in hospitals [1]. It is imperative to diagnose acute aortic dissection (AAD) in the emergency room, as within the first 48 hours, each hour of intervention delay raises the mortality rate by 1-2% [2]. The Stanford classification system divides thoracic aortic dissection into two types: type A involves dissection of the ascending aorta, while type B involves dissection of the descending aorta (beyond the origin on the left subclavian artery). Mortality is higher in Stanford type A compared to type B [1].

Classically, aortic dissection presents as severe pain in the chest, back, or abdomen that appears suddenly. A typical presentation, on the other hand, might have serious repercussions due to a late diagnosis [2]. Coronavirus disease 2019 (COVID-19) increases the risk of many cardiovascular problems, including acute myocardial damage, rhythm disturbances, cardiogenic shock, acute coronary artery disease, and venous thrombosis [3].

Aortic dissection is an important but often underestimated issue in COVID-19 patients, despite a few articles indicating an increase in incidence during the pandemic [4]. Our case report highlights the importance of recognizing the atypical presentation of a potentially fatal aortic dissection, especially during the COVID-19 pandemic, as it can distract emergency physicians. We present a case of silent Stanford type-B aortic dissection accidentally discovered during the evaluation of a COVID-19 patient, which was managed conservatively.

## Case presentation

**Figure 1:**
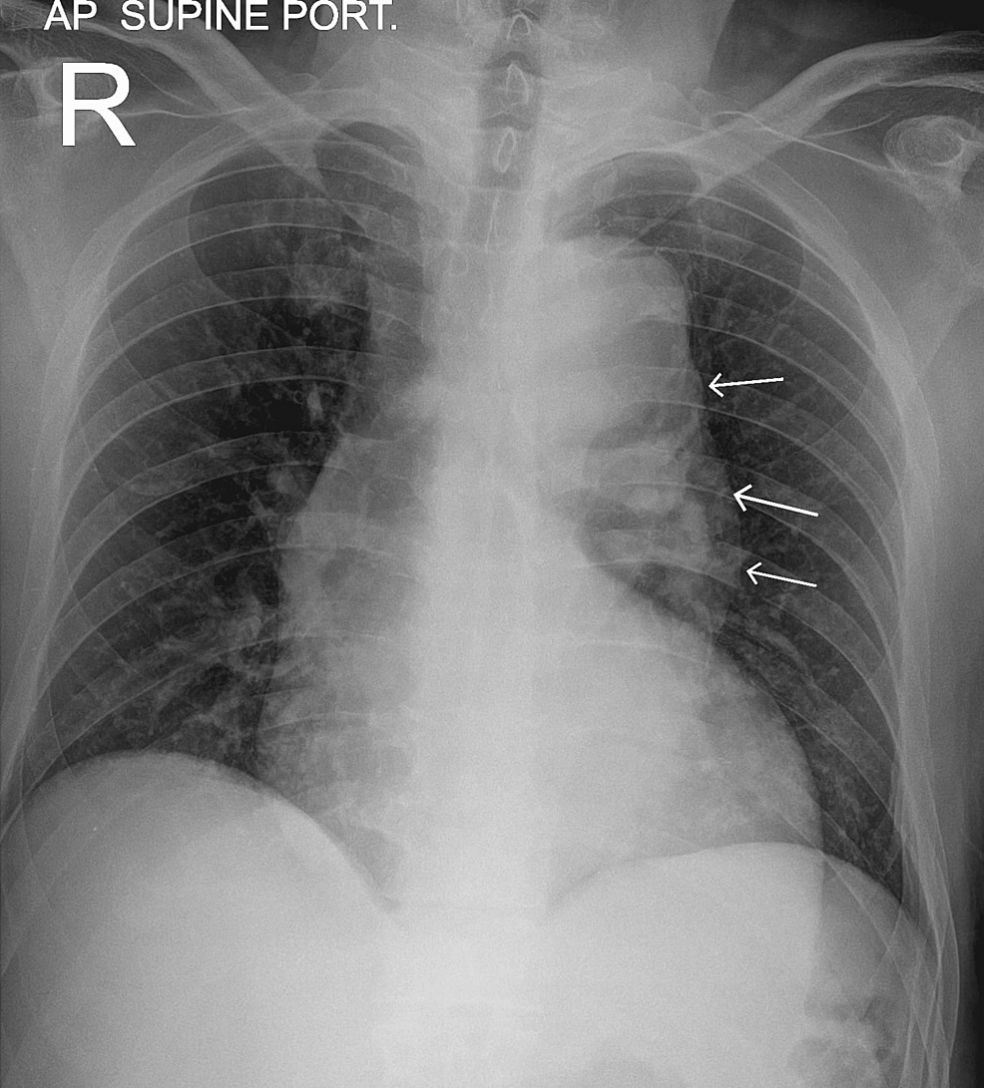
X-ray chest showing wide mediastinum (white arrows)

**Figure 2:**
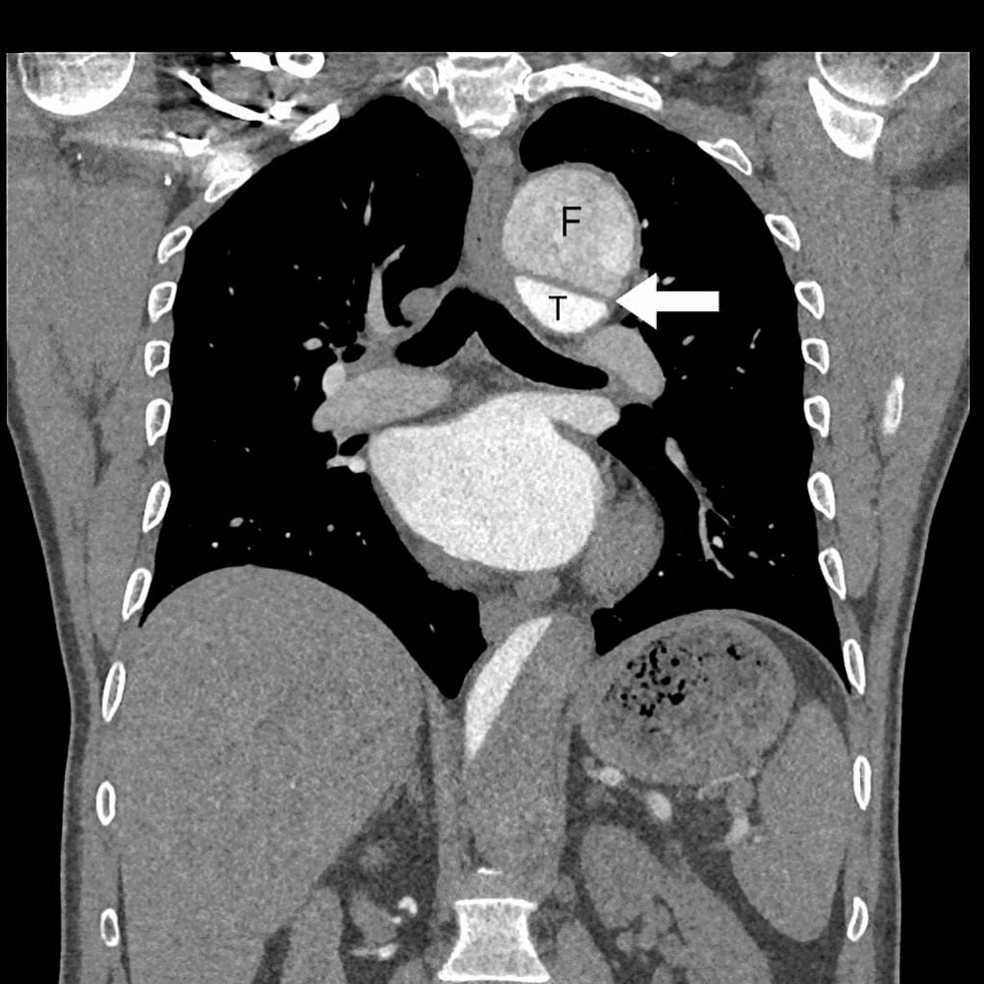
CT angiogram of the chest, coronal view, demonstrating an intimal flap (white arrow) in the descending aorta with the formation of true and false lumens

A previously healthy 55-year-old male patient, a nonsmoker with no comorbidities, arrived at the emergency department with a three-day history of fever, cough, and dyspnea. Patient was diagnosed to be COVID-19 positive on home isolation. On examination, the patient was hypertensive (160/115 mmHg), had a heart rate of 98 beats per minute, a respiratory rate of 16 breaths per minute, a temperature of 36.2 °C, and an oxygen saturation of 99.9%. D-dimer was 6 mg/L (normal value less than 0.5 mg/L). The X-ray chest didn't show any pulmonary infiltrates but showed a wide mediastinum (Figure 1). Computed tomography (CT) of the chest was done to rule out pulmonary embolism and assess for possible aortic dissection. On CT, his chest revealed a severe aortic dissection (Figure 2), with true and false lumens visible arising from the thoracic aorta slightly distal to the origin of the left subclavian artery and continuing inferiorly up to the origin of the renal arteries. Both the true and false lumens of the aortic dissection are noted to be opacified, with opacification of the true lumen occurring prior to the false lumen. The true lumen is noted to be narrowed and compressed by the false lumen. The larger false lemon is noted along the left posterior-lateral aspect of the thoracic aorta. The descending thoracic aorta is consequently markedly dilated and ectatic secondary to dissection, measuring up to 5.2 cm. Repeated inquiries to the patient about the symptoms of the dissection were futile. The patient's echocardiography revealed a normal heart with a decent ejection fraction and normal valves. He was hospitalized with a COVID-19 infection as well as a Stanford type-B aortic dissection. The patient started on a labetalol infusion starting with 30mg hourly, amlodipine 5mg once daily, and enoxaparin 70mg twice daily for subcutaneous and symptomatic treatment for COVID-19 infection. The patient improved with symptomatic therapy for fever, cough, and blood pressure control. The cardiothoracic surgery team decided on conservative treatment for a non-complicated Stanford type-B aortic dissection. Patient was discharged home after five days of uneventful observation with outpatient follow-up. He had a second CT scan, which showed no change in the size of the dissection.

## Discussion

We report the incidental discovery of a silent Stanford type-B aortic dissection on CT scan assessment of a patient with COVID-19 infection. The exact timing of the development of the aortic dissection is unknown, as there were no initial suspicions of this condition. The patient did not present with typical symptoms such as backache, chest pain, loss of consciousness, or abdominal pain.

Aortic dissection is a form of acute aortic syndrome that mostly affects males (2:1) and people in their sixth and seventh decades of life [5]. It is linked to a variety of risk factors, including high blood pressure, cigarette smoking, and genetic conditions including Marfan syndrome (MFS) and Ehlers-Danlos [6]. Additionally, conditions like Takayasu arteritis, Behcet's disease, and syphilis infection can contribute to the development of aortic dissection [7]. Studies have also shown an increased incidence of aortic dissection during the first wave of COVID-19, as well as other cardiovascular conditions such as cardiac infarction and aneurysms of the aorta [5]. According to some studies, COVID-19 infection may down-regulate angiotensin-converting enzyme 2 and activate the renin-angiotensin-aldosterone pathway, resulting in hypertension that may contribute to aortic dissection [8]. Molecular pathways and markers shared by aortic dissection and COVID-19 have also been identified, including elevated levels of Von Willebrand Factor (VWF) [9] and various molecular factors associated with both conditions, such as transforming growth factor beta (TGF-β), total plasma homocysteine Y (tHCy), and matrix metalloproteinases (MMPs) [10-12].

Traditionally, an aortic dissection without pain was considered rare. However, recent information indicates that painless aortic dissection is a clinical variant that can present with symptoms related to complications arising from the dissection [13]. Classic symptoms of tearing chest, back, or abdominal pain may be absent in approximately 10% of patients [13-15]. Syncope, new-onset neurological deficits, congestive cardiac failure, strokes, spinal cord ischemia or coma, acute kidney failure, cardiac infarction, and ischemia of the mesenteric vessels have all been linked to the presentation of painless aortic dissection, according to the International Registry of Acute Aortic Dissection (IRAD) [16].

In our patient, together with the absence of pain, the patient did not exhibit any of these associated features. He denies any syncope, had a normal neurological exam, and echocardiography revealed a normal heart with a decent ejection fraction, normal valves, and normal kidney functions. Instead, he presented with mild symptoms of the COVID-19 infection.

At the time of diagnosis, the patient was stable and did not show any signs of complications, such as refractory hypertension, poor perfusion, or partial false lumen thrombus. The overall aortic diameter was less than 55 mm [17]. The cardiothoracic surgery team decided to pursue a conservative treatment approach and continue regular follow-up with repeat imaging at three months, nine months, and then annually.

Patient follow-up at a three-month interval showed controlled blood pressure and no pain, and a second CT scan showed no change in the size of the dissection.

## Conclusions

Aortic dissection is a critical condition that poses a significant risk to life and health. The characteristic symptom of intense pain in the chest, back, or abdomen may be absent. It can also present with complications of dissection such as syncope, myocardial infarction, a new neurological deficit, and acute kidney injury, or it can also be totally silent and accidentally discovered, as shown in our case. It is important for the emergency physician to maintain high suspicion, which helps him identify the life-threatening conditions, including aortic dissection, that started to present atypically, particularly during COVID-19.
